# Monthly monitoring of suspended sediment variability using bottle samplers in the LUSI outfall area of the Porong River

**DOI:** 10.1016/j.mex.2025.103592

**Published:** 2025-08-28

**Authors:** Rossana Margaret Kadar Yanti, Ria Asih Aryani Soemitro, Mahendra Andiek Maulana, Trihanyndio Rendy Satrya, Dwa Desa Warnana, Moh Muntaha

**Affiliations:** a Institut Teknologi Sepuluh Nopember, Department of Civil Engineering, Sukolilo, Surabaya, 60111, Indonesia; b Institut Teknologi Sepuluh Nopember, Department of Geophysical Engineering, Sukolilo, Surabaya, 60111, Indonesia; c Institut Teknologi Sepuluh Nopember, Department of Infrastructure Engineering, Sukolilo, Surabaya, 60111, Indonesia; d Institut Teknologi Kalimantan, Department of Civil Engineering and Planning, Karang Joang, Balikpapan, 76127, Indonesia

**Keywords:** Alluvial river sediment, Field-based monitoring, Field validation, Mud volcano, Multi-depth sampling, USDH 48-type bottle samplers

## Abstract

Monitoring suspended sediment concentration (SSC) is important for understanding sediment dynamics in extreme tropical rivers influenced by both natural and human activities. The Porong River in East Java constantly receives sediment input from LUSI (short for Lumpur Sidoarjo), a hot mud volcano formed after a drilling incident at Banjar Panji-1 in May 2006. The eruption inundated over 6.3 km² of settlements, farmland, and infrastructure, displacing about 30,000 people and causing persistently high sediment loads with extremely turbid flows. This study developed a field-based SSC sampling method by modifying the USDH-48 bottle, replacing standard rods with flexible ropes for manual operation of orientation and depth in deep, high-energy flows. The method uses a systematic spatial-vertical sampling strategy across several cross-sections to capture sediment variability. In contrast to ASTM D3977–97, which is designed mainly for shallow, steady-flow rivers, this method accommodates greater depths and unsteady-flow rivers through flexible deployment and structured sampling coverage. Over one year, monthly monitoring produced 864 samples. Validation against a messenger-system vertical sampler at the same location and depth showed strong statistical agreement (R² > 0.80; RMSE < 20 %).

Adaptive field methods based on the modification of USDH-48 for extreme tropical rivers.

Structured design capturing spatial and vertical sediment variability.

Statistically validated against an established vertical water sampling system.

## Graphical abstract

**Figure:**
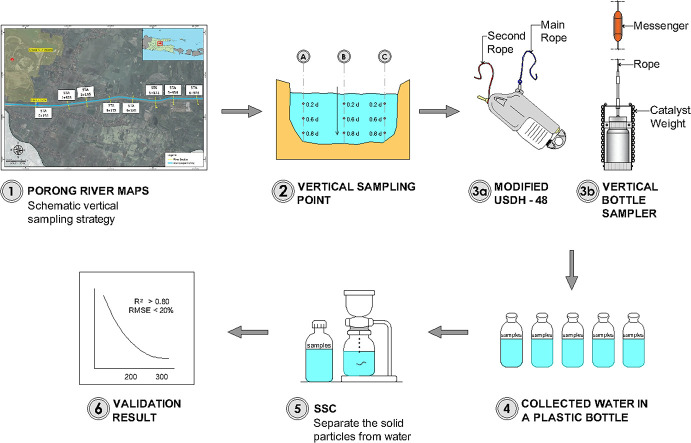

Specifications table

*This table provides general information on your method.*

**Table:** 

**Subject area**	Environmental Science
**More specific subject area**	*Alluvial river sediment with additional sediment sources*
**Name of your method**	*Modified Multi-Depth Sediment Sampling Method with USDH 48-type bottle samplers in Stratified River Flow*
**Name and reference of original method**	*Adapted from ASTM D3977–97 with field-based modifications for high-sediment tropical rivers*
**Resource availability**	*Not available*

## Background

Monitoring suspended sediment concentration (SSC) is an essential parameter in river management, hydrological studies, and assessing water quality. SSC data is important for understanding erosion and sedimentation processes, as well as the transport of sediment and pollutant materials [1,2]. In tropical regions, especially those influenced by geological and anthropogenic activities, sediment dynamics are highly fluctuating and complex both spatially and temporally [3].

The Porong River in East Java, Indonesia, is an extreme example of a dynamic tropical river system. Since 29 May 2006, the LUSI (short for Lumpur Sidoarjo) mud eruption, initiated after the Banjar Panji-1 drilling incident, has discharged massive volumes of volcanic mud into the Porong River via the Ginonjo outfall [4]. The eruption inundated over 6.3 km² of settlements, farmland, and infrastructure, displaced around 30,000 people, and caused persistently high sediment loads with extremely turbid flows [5,6]. River depth now reaches 9 m, with hydrodynamics affected by seasonal variations and tides [[7], [8], [9]].

The discharge of the Porong River ranges from approximately 45 m³/s in the dry season to >2500 m³/s during the wet season [8]. Under such extreme changing hydrodynamic conditions, conventional SSC sampling presents significant challenges. Standard methods, like ASTM D3977–97, focus on laboratory procedures and are typically applied to shallow, steady-flow rivers, including the Calapooia River (Oregon) and the Ulla River (Spain). The USDH-48 isokinetic sampler—commonly used for depths under 3 m and moderate flows—is constrained by weight, capacity, and nozzle stability in deep, high-energy tropical rivers [[10], [11], [12]]. These limitations risk underestimating sediment concentrations in the deeper water column, a critical layer for accurate total load estimation. In the case of the Porong River, a method must be able to operate at depths exceeding 9 m, withstand unsteady currents, and collect representative samples from multiple depths and locations along the river.

A limitation identified in earlier studies is the lack of explicit reporting on spatial and vertical strategies in the SSC sampling design. A study by Groten & Johnson in 2018 only sampled SSC at one cross-section point without depth variation [13]. In 2024, Vázquez-Tarrío created a seasonal approach in the Ulla River but did not report the depth distribution or its vertical replication [14]. Pomázi and Baranya only used one type of sampler in 2020, and they did not have a reference instrument, so it was impossible to compare the accuracy of different instruments [15]. This situation highlights the importance of flexible, organized, and easy sampling methods for suspended sediment concentration (SSC), particularly in tropical rivers with challenging conditions. By introducing a modified USDH-48 with structured spatial–vertical coverage, this study provides a method capable of operating in deep, high-energy tropical rivers while addressing the reporting and validation gaps in previous research. The approach complements existing SSC protocols and expands their applicability to environments that current international standards have not addressed. To fill these gaps, the objective of this study is to develop an effective field-based SSC sampling method in extreme tropical rivers.

## Method details

**Fig. 1:**
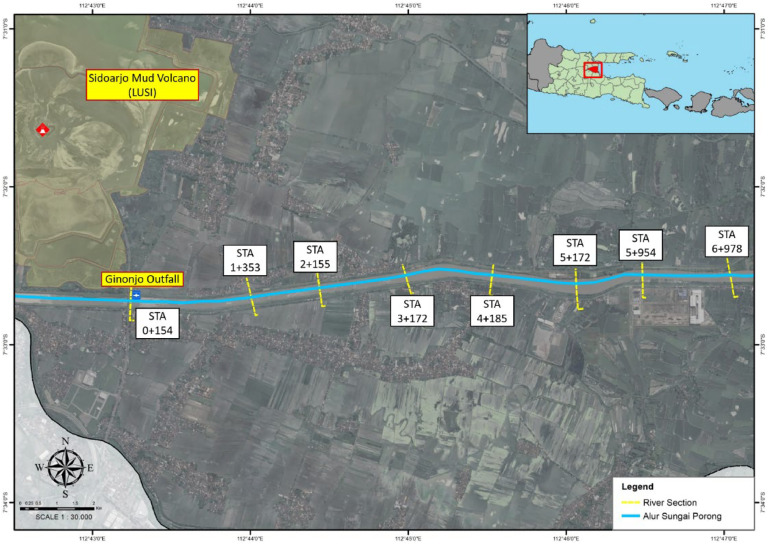
Research Study Location. (Source: Authors modified from Google Earth).

The method for sampling suspended sediment concentration (SSC) was conducted in the Porong River, East Java, Indonesia, which has been receiving volcanic mudflow from the LUSI (Lumpur Sidoarjo) Ginonjo outfall since 2006. The SSC sampling procedure began at the cross-section upstream of the Ginonjo outfall and extended approximately 7 km downstream. Samples were taken at eight cross-sections, each approximately 1 km apart. Sampling began at STA 0 + 154, which is 154 m from the Ginonjo outfall (STA 0 + 000), and continued up to STA 6 + 975 (Fig. 1).

**Fig. 2:**
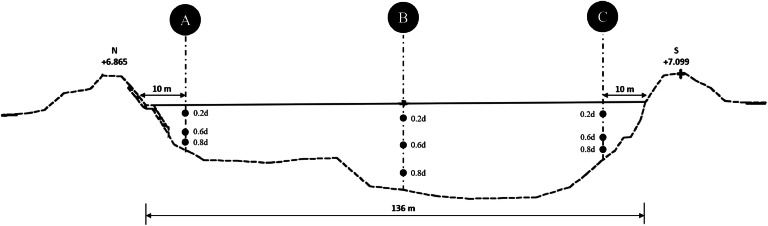
Cross Section of the Porong River at STA 0 + 154. (Source: Perum Jasa Tirta (PJT) 1).

At each station (STA), samples are collected from three water columns along the river: the left bank (A), the middle (B), and the right bank (C). Each water column is divided into three vertical depths: 0.2d (near the surface), 0.6d (middle of the water column), and 0.8d (near the bottom), with d being the total river depth measured using a gauge board (Fig. 2). The depth sampling method in this study is based on the concept of vertical distribution, specifically the Equal-Width Increment (EWI) and Equal-Depth Increment (EDI) methods, which are generally used to sample sediments in open waters [2,16,17]. However, these approaches are not explicitly designed for deep, highly turbid tropical river conditions that are difficult to access. Therefore, these basic principles were operationally adapted into depth-proportional procedures (0.2d, 0.6d, and 0.8d) that can be performed from a boat. This procedure enables the acquisition of consistent vertical data in rivers with depths greater than 9 m, even under limited field conditions, providing a practical alternative for tropical river systems that are not yet covered by conventional standards. This depth interval helps in understanding the vertical distribution and obtaining comprehensive data on the concentration of suspended sediments in the water column [[18], [19], [20]].

**Fig. 3:**
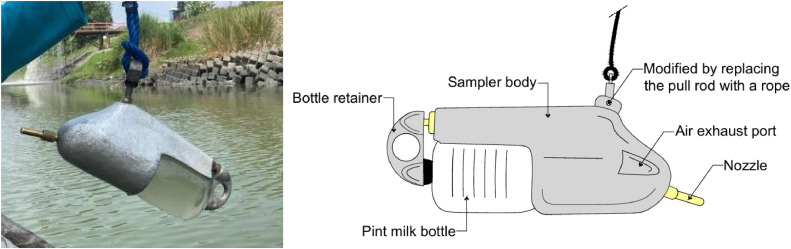
USDH 48-type bottle samplers with flexible ropes replacing the standard rods. (Source: Authors).

**Fig. 4:**
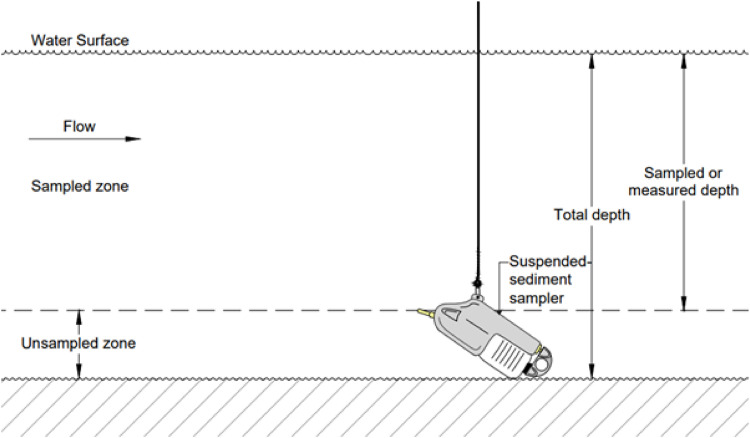
An illustration of suspended sediment concentration sampling using USDH-48. (Source: Authors).

Samples were taken using USDH-48 type bottles (without form modification) but provided with flexible ropes replacing the standard rods to allow operation at depths exceeding 9 m from the boat (Fig. 3). This bottle has a maximum volume of 470 mL [10], but is only used in the range of 375–420 mL to avoid bias due to overfilling. The USDH-48 is set up horizontally, facing upstream (Fig. 4). With this orientation, the nozzle is manually operated using two ropes from the boat to keep it in line with the flow. The main rope (vertical rope) is used to lower and raise the sampler vertically from the boat. Control rope (horizontal/side rope) attached to the back or side of the sampler (usually near the bottom or tail of the bottle) and pulled from the boat to control the nozzle's orientation. The nozzle orientation is maintained facing the main current (upstream) by utilizing control ropes from the boat, based on visual guidance by the operator. Although not as precise as a standard rod, this control proved sufficient to maintain a relatively stable horizontal position in most flow conditions.

Before sampling, the current velocity was measured using a Valeport 106 current meter (Valeport Ltd., UK) at each depth point (0.2d, 0.6d, and 0.8d). This velocity data is used to determine the immersion time required for the water to fill the bottle within the effective volume range (10 s average per point), based on the assumption of an incoming flow approaching isokinetic conditions [21]. This immersion time is not determined by visual trial and error but calculated from the average flow rate measured at the relevant depth points, as well as the target bottle volume (375–420 mL), assuming the incoming velocity approaches isokinetic conditions.

**Fig. 5:**
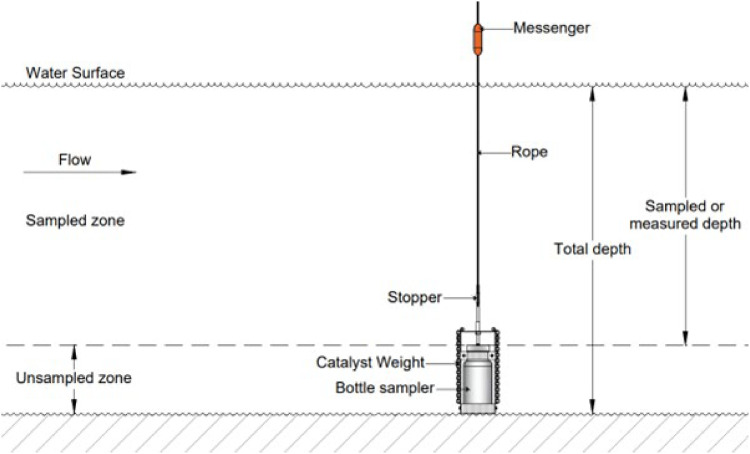
An illustration of suspended sediment concentration sampling using a vertical water sampler. (Source: Authors).

Each observation point was replicated twice to ensure the consistency of the results. One additional sample was collected using a messenger-based vertical water sampler with a 500 mL tube volume at the same location and depth for method validation objectives. The instrument was lowered vertically to the target depth, then allowed to stabilize before being dropped with a messenger to close the valve and capture the water. After the valve was closed, the device was raised to the surface for sample analysis (Fig. 5). This inter-instrument validation strategy refers to common practices in comparative studies of sampling instruments [13,15].

**Fig. 6:**
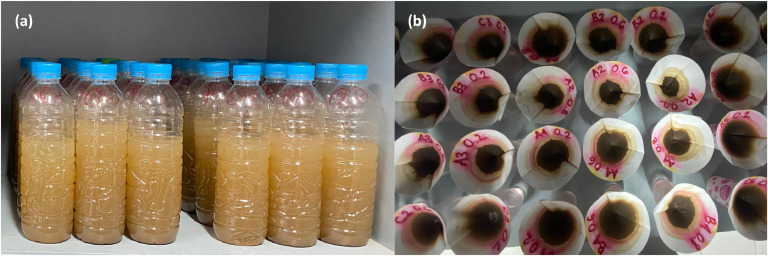
Laboratory analysis: a) the collected water was transferred to a plastic bottle labeled with the corresponding STA and location code; b) water was filtered through Whatman No 42 filter paper to separate the solid particles from the water. (Source: Authors).

The sampled water is immediately transferred to a clean, labeled plastic bottle with a code according to the STA and then taken to the laboratory for analysis using the filtration method specified in ASTM D3977–97 (Fig. 6). The average volume of the samples analyzed was 400 mL. The sample was allowed to stand until the sediment settled. Following ASTM D3977–97, Procedure B, the clear upper portion of the supernatant was carefully decanted to avoid disturbing the sediment layer, leaving approximately 50 mL of water above the settled sediment. The remaining portion, which contains concentrated suspended particles, was filtered using Whatman No 42 filter paper with a pore size of 2.5 µm. This kind of filter is effective at separating fine particles, such as clay and silt [22], which are dominant in the suspended sediment fraction of the Porong River. The weight of the sediment was obtained from the difference in the mass of the filter before and after drying, and the concentration result was expressed in mg/L according to the formula:

(1) $$SSC\;\left(mg/L\right)=\frac{\left(W_{t}-W_{f}\right)}{V}$$

Where:

Wt = weight of the filter paper plus dried sediment (grams)

Wf = the initial weight of the empty filter paper (grams)

*V* = volume of the water sample filtered (milliliters, ml)

The entire method was carried out monthly over 12 months, from January to December 2023, to account for seasonal variability in suspended sediment concentration (SSC) during both the wet and dry seasons. While the timing was not directly aligned with the tidal cycle, data collection was consistently scheduled at the same hour each month to ensure temporal consistency.

## Method validation

### Validation design and objectives

The method validation process in this study is designed with reference to the framework of previous research using USDH-48 and similar instruments for measuring suspended sediment concentration (SSC) in rivers [2,10]. However, this study extends the process through the systematic development of spatial-vertical validation and the application of the margin of error (MoE) as a quantitative indicator of precision, as well as the adaptation of methods to the typical field challenges in deep, turbid, and unreachable tropical river systems.

Unlike previous studies that generally focused on subtropical rivers with open terrain, this method is applied in tropical environments with extreme hydrodynamic characteristics and high levels of sediment heterogeneity. Validation was conducted in three water columns (left bank, middle, and right bank) and three depth layers (0.2d, 0.6d, and 0.8d) at eight cross-sections, providing more detailed spatial and vertical information.

The main objective of this validation is to ensure that the sampling method accurately reflects the amount of suspended sediment in the river and complies with scientific standards, as outlined in ASTM D3977–97, ISO 4365 [23], and TWI 3-C2 [10].

### Location, time, and sampling procedure

Validation was conducted over two months, specifically in May and June 2023; however, only data from June are used in this manuscript, as more complete data were available for that month. The validation locations consist of eight cross-sections along the Porong River (STA 0 + 154 to STA 6 + 978). Sampling was carried out at each cross-section across three water columns: the left bank (A), the middle (B), and the right bank (C). Samples were taken from each column at three depths: 0.2d, 0.6d, and 0.8d, which are equal to the total depth (d), for a total of 72 validation points.

Samples were taken simultaneously from the same location and depth using two types of instruments. USDH-48 was used without any modifications to its physical form, but its operating system was replaced with a flexible rope. The horizontal position of the nozzle is manually adjusted from the boat to align it with the main current. The effective volume of this tool ranges from 375 to 420 mL. For comparison, a 500 mL capacity vertical water sampler based on a messenger system was used. The instrument was lowered vertically to the target depth, then allowed to stabilize before being dropped with a messenger to close the valve and capture the water. After the valve was closed, the device was raised to the surface for sample analysis. Each observation point was replicated twice using the USDH-48, with the SSC value analyzed as the average of the two. Sampling using a vertical water sampler was conducted once as a reference. The entire process is carried out at the same time every month to maintain temporal consistency, although it is not specifically aligned with the tidal cycle.

### Statistical analysis and evaluation of the results

An evaluation was conducted between the modified USDH-48 method and the vertical water sampler as a reference, using three main statistical parameters: coefficient of determination (R²), root mean square error (RMSE), and a two-tailed paired *t*-test. The coefficient of determination (R²) is used to measure how well the reference sampler can explain the variability of SSC from USDH-48. The validation results showed R² values ranging from 0.875 to 0.993. The best consistency was observed in the middle column (B) at all depths, specifically R² > 0.98, indicating a very strong linear correlation between the two methods. This value shows that the USDH-48 measurement results are generally consistent and follow the sediment distribution pattern captured by the reference method, in line with similar findings in field studies by Hutasuhut [24] and Pomázi & Baranya [15], which demonstrated R² ≥ 0.9 as a high accuracy point for SSC measurements in rivers.

Root mean square error (RMSE) is defined as a percentage, showing the average deviation from the reference value and assessing the quantitative error. The RMSE values in this study are all below 20 %, indicating that the levels of measurement error are relatively low and still acceptable for field surveys that involve a lot of variation. For example, the lowest RMSE value of 4.88 % in column B–0.2d means that the data is very accurate. On the other hand, the highest RMSE value (17.21 %) in column C–0.6d indicates that there are greater differences due to local turbulence and changes in flow at the riverbank. RMSE values <20 % indicate a deviation level that is still acceptable for estimating suspended sediment in rivers with complex morphology [25].

The paired *t*-test was also used in this study to find out the SSC difference between the USDH-48 method and the reference. The p-values are mostly <0.05, but these differences are just numbers and don't show a consistent or systematic bias because the values change from column to column and depth to depth. This p-value indicates that the differences between the two methods are mainly due to variations in hydrodynamics and sediments. These results support the conclusion that USDH-48 remains a feasible option for the scientifically valid sampling of SSC. Table 1 shows the R² values, RMSE, and p-values from the paired *t*-test for each combination of water column and depth.

**Table 1:** Validation results comparing USDH-48 and vertical water sampler, organized by water column and depth.

Water Column	Depth	R²	RMSE	p-value
			(%)	
A	0.2d	0.875	12.98	0.005
A	0.6d	0.896	14.66	0.014
A	0.8d	0.912	15.28	0.027
B	0.2d	0.981	4.88	0.0005
B	0.6d	0.993	5.38	0.001
B	0.8d	0.918	11.23	0.016
C	0.2d	0.981	17.08	0.008
C	0.6d	0.993	17.21	0.006
C	0.8d	0.918	16.27	0.027

(Source: Authors).

The highest accuracy was found in column B, which has a more stable flow. In contrast, larger deviations occurred in columns A and C, especially at a depth of 0.8d This difference was caused by local turbulence, the change in the USDH-48 nozzle direction when controlled by a rope, and the difficulty in maintaining isokinetic conditions due to changes in microflow velocity. Furthermore, differences in the operating principles of the instruments contributed to this issue: the vertical water sampler captures water in a closed mode. Closed mode means that water is captured and sealed at a specific depth, preventing it from mixing with water from other layers. At the same time, the USDH-48 relies on passive inflow, which is more sensitive to orientation and local current conditions.

### Assessment of accuracy and variability of results

To assess the accuracy and reliability of the SSC sampling method using the modified USDH-48, this study examined three statistical measures: the margin of error (MoE), the limits of agreement (LoA) from the Bland–Altman analysis, and the coefficient of variation (CV %). All of the parameters provide an overview of the measurement area, addressing accuracy, method agreement, and replication consistency. The margin of error (MoE) is calculated based on the average difference between two SSC measurements taken at the same point using two different instruments: the modified USDH-48 and the vertical water sampler, which serve as a comparison. MoE represents the level of precision of the measurement results under similar conditions and locations, as well as the instrument's sensitivity to local variability.

The MoE values obtained ranged between ±1.34 and ±14.29 mg/L. The smallest MoE was found in the water column B–0.2d (±1.34 mg/L from SSC 73 mg/L), indicating that in the main flow area with relatively stable hydrodynamics, both instruments provided very similar results. The highest MoE was found in column C–0.8d (±14.29 mg/L from SSC 139 mg/L), indicating greater differences between methods near the riverbed, where turbulence and complex sediment distribution occur [12].

The MoE ranges from 1.8 % to 10.3 % of the average SSC value, in relative terms. Variations of up to 10 % between methods are still acceptable in the context of field measurements in rivers with high dynamics [14,17]. The MoE values suggest that the SSC measurement results obtained from the modified USDH-48 are within acceptable limits; however, they still require monitoring in high-flow areas.

The Bland–Altman analysis was used to assess whether the SSC results from both methods can be interchangeable. Narrow LoA indicates that the differences between methods do not contain significant systematic bias. The results show that the narrowest LoA is at B–0.2d (±3.13 mg/L), while the widest LoA is at C–0.8d (±33.50 mg/L). This range indicates that the validity of the methods is highly dependent on flow dynamics and depth. The narrowest LoA range is achieved at B–0.2d (±3.13 mg/L), indicating that there is no significant systematic bias between methods at this location. The highest variability is observed at C–0.8d, with a range of ±33.50 mg/L. This value indicates a larger difference between methods in the lower part of the water, which is influenced by local turbulence, interactions with sediment on the bottom, and unstable flow patterns [13,14].

The coefficient of variation (CV %) is used to measure the precision between replicates at each SSC measurement point with the modified USDH-48. The results show that all CV % values are below the 12 % threshold as specified in ISO 11,352:2012 for surface water precision testing.

The highest CV % value (>11 %) was recorded at a depth of 0.8d in columns A and C, indicating increased suspense in the transition zone near the riverbed. Conversely, the lowest CV % values (<6 %) were observed at B–0.2d and B–0.6d, indicating that the main flow center is relatively stable and delivers the most precise results. This condition is consistent with the results of Horowitz and Jalón-Rojas, which show that lateral and vertical SSC variation is very high in tropical rivers with non-uniform currents.

Details of the Margin of Error (MoE) and Limits of Agreement (LoA) values are presented in Table 2, and the Coefficient of Variation (CV %) for each combination of water column and depth is presented in Table 3.

**Table 2:** The Margin of Error (MoE) and Limits of Agreement (LoA) Values.

Water Column	Depth	Average SSC	Range LoA	LoA Width
		(mg/L) ± MoE (95 % CI)	(mg/L)	(mg/L)
A	0.2d	80 ± 5.25	8.39 ± 12.30	20.69
A	0.6d	124 ± 10.70	14.22 ± 25.08	39.30
A	0.8d	109 ± 10.30	11.83 ± 12.33	24.16
B	0.2d	73 ± 1.34	3.29 ± 3.13	6.42
B	0.6d	112 ± 2.20	4.87 ± 5.16	10.03
B	0.8d	149 ± 9.89	12.74 ± 23.2	35.94
C	0.2d	61 ± 5.78	8.58 ± 13.54	22.12
C	0.6d	113 ± 9.84	15.49 ± 23.07	38.56
C	0.8d	139 ± 14.29	16.34 ± 33.50	49.84

(Source: Authors).

**Table 3:** The Coefficient of Variation (CV %) for each combination of water column and depth.

STA	Depth	Water Column	CV	Water Column	CV	Water Column	CV
(m)			(%)		(%)		(%)
0 + 154	0.2	A	6.36	B	6.01	C	7.42
1 + 353	0.2	A	7.78	B	7.00	C	8.42
2 + 155	0.2	A	9.95	B	7.11	C	8.53
3 + 172	0.2	A	9.85	B	6.57	C	7.98
4 + 185	0.2	A	6.40	B	5.71	C	7.14
5 + 172	0.2	A	10.99	B	8.04	C	9.46
5 + 954	0.2	A	7.00	B	3.48	C	4.90
6 + 978	0.2	A	10.83	B	6.59	C	8.00
0 + 154	0.6	A	8.34	B	4.10	C	5.51
1 + 353	0.6	A	8.30	B	4.73	C	6.14
2 + 155	0.6	A	4.26	B	5.69	C	7.11
3 + 172	0.6	A	8.56	B	7.15	C	8.56
4 + 185	0.6	A	5.88	B	4.87	C	6.28
5 + 172	0.6	A	5.24	B	3.83	C	5.24
5 + 954	0.6	A	8.68	B	6.59	C	8.01
6 + 978	0.6	A	5.65	B	4.24	C	5.65
0 + 154	0.8	A	8.49	B	7.07	C	9.90
1 + 353	0.8	A	7.82	B	7.11	C	8.52
2 + 155	0.8	A	10.68	B	9.98	C	11.39
3 + 172	0.8	A	10.53	B	9.89	C	8.48
4 + 185	0.8	A	9.19	B	8.49	C	11.31
5 + 172	0.8	A	9.29	B	9.91	C	11.33
5 + 954	0.8	A	9.80	B	9.80	C	11.22
6 + 978	0.8	A	8.75	B	8.75	C	11.58

(Source: Authors).

By combining these three indicators, the validation results show that the USDH-48 modification not only produces accurate SSC estimates (as indicated by R² and RMSE) but also demonstrates precision (CV %) and reliability across methods (LoA). This method provides a comprehensive evaluation framework for addressing the challenges of collecting suspended sediment data in fluctuating and dynamic tropical rivers. This result enhances the validity of the method from both technical and practical viewpoints.

### Discussion on method validation

The validation of the method reveals that the suspended sediment concentration (SSC) values obtained from the modified USDH-48 show a strong linear relationship with those from the vertical sampler, serving as the reference method, with R² values ranging from 0.875 to 0.993. The highest values were consistently found in the middle column (B), which hydrodynamically functions as the mainstream flow direction, where the distribution of SSC is more uniform and representative. These findings support Gray's study, which states that the distribution of SSC in the middle column generally provides the closest estimate to the average cross-section value in an open channel [26].

Evaluation based on R² and Root Mean Square Error (RMSE) is not enough to fully describe the uncertainty of the method. Therefore, additional methods, such as the Margin of Error (MoE), Limits of Agreement (LoA), and paired *t*-test analysis, were applied. The results show no significant difference between the two methods (*p* > 0.05), and the MoE ranges from ±8–12 %, approaching the field measurement standards as specified in ISO 11,352:2012 and USGS sediment protocols [21,27].

In addition to accuracy, the precision between replicates using USDH-48 was also tested using the coefficient of variation (CV %). The CV values were mostly <10 %, especially at a depth of 0.6d and column B, indicating high reproducibility of results in the main river flow zone. These results are in line with Horowitz's research, which identified that the largest vertical variations in SSC generally occur near the riverbed and surface due to the influence of local turbulence, bed sediment interaction, and mixing [17].

The vertical distribution of SSC in the Porong River shows an increase in concentration near the riverbed, consistent with the distribution pattern in tidal-influenced tropical river systems. This result is in line with what Syvitski found, which explains that tropical rivers with high sediment supply and seasonal discharge variation tend to exhibit a vertically non-homogeneous SSC profile due to fluctuations in turbulence energy, backwater effect, and density stratification [28].

The results show a good level of accuracy, but there are still some limitations. The use of a flexible rope on the USDH-48 may lead to angular deviation and potentially underestimate micro-sediment volume in turbulent currents, resulting in sampling bias [12]. Also, validation was carried out over a brief two-month period (May and June), which is inadequate to capture the full seasonal variability commonly found in tropical regions, including wet and dry seasons. These limitations are particularly significant, given that the LUSI mudflow, the discharge of the Brantas River, and daily tidal fluctuations considerably impact the supply and distribution of sediment in the Porong River.

### Validation conclusion: A summary of findings and implications

Validation shows that the modified USDH-48 is capable of producing accurate, precise, and comparable SSC estimates to those obtained with the vertical water sampler. High R² values (0.875–0.993) and MoE < 12 % indicate the consistency of results between methods, with no statistically significant differences (*p* > 0.05). CV % values between replicates <10 % show good accuracy, especially in the middle column (B) and at a depth of 0.6d

The increased vertical distribution of SSC near the bottom was successfully captured, reflecting the effectiveness of the spatial-vertical approach used. Although the validation was limited to two months, these results suggest that the modified USDH-48 is a suitable alternative method for monitoring SSC in dynamic tropical rivers, demonstrating adequate efficiency and precision.

In general, this approach provides a practical and affordable solution for long-term studies in complex tropical regions. This study helps close the gap between the precision of lab work and the reality of work in the field.

## Limitations

This study has a few limitations that need to be noted. First, the method validation was only conducted in May and June, when flow conditions were relatively stable. Its effectiveness under extreme conditions, such as flood seasons or short-term high discharge events, is still untested. Second, the monthly sampling rate might fail to catch short-term SSC peaks due to flash floods or abrupt sediment discharges. These events may happen between established sampling dates, and their short-lived but intense SSC peaks can significantly influence the overall SSC trend. Consequently, the capability of monthly sampling to represent long-term conditions is limited, especially in a river with highly fluctuating flows such as the Porong. To better represent long-term dynamics, future studies should consider a combination of higher-frequency sampling and historical observations across different flow regimes (stable, high, and fluctuating) over multiple years. Third, despite the spatial–vertical coverage, extreme field conditions in the Porong River, such as depths exceeding 9 m, strong currents, and heterogeneous sediment distribution, may still affect sample representativeness. Fourth, the approach depends on manual control of the USDH-48 nozzle orientation via a flexible rope. This method may lead to discrepancies and hinder genuine isokinetic conditions in turbulent or deep water flows.

Future studies should broaden validation efforts to encompass a more diverse array of hydrological scenarios, particularly high flow events. Enhancing nozzle control through directional weights or an angle positioning system, alongside the integration of sensors or lab tests for objective isokinetic evaluation, may also increase method reliability.

## Ethics statements

Not applicable.

## Related research article

None.

## CRediT author statement

**Rossana Margaret Kadar Yanti:** Conceptualization, Methodology, Validation, Writing – original draft. **Ria Asih Aryani Soemitro:** Supervision, Writing – review & editing, Final approval of the manuscript. **Mahendra Andiek Maulana:** Supervision, Writing – review & editing. **Trihanyndio Rendy Satrya:** Supervision, Writing – review & editing. **Dwa Desa Warnana:** Supervision, Writing – review & editing. **Moh Muntaha:** Supervision, Writing – review & editing.

## Declaration of competing interest

The authors declare that they have no known competing financial interests or personal relationships that could have appeared to influence the work reported in this paper.

## Data Availability

Data will be made available on request.
